# Supplementary material for: Consumer preferences, experiences, and attitudes towards telehealth: Qualitative evidence from Australia
Source: PLoS One. 2022 Aug 31;17(8):e0273935. doi: 10.1371/journal.pone.0273935 (PMC9432716; doi:10.1371/journal.pone.0273935)
Supplement: S1 File — (PDF) [file pone.0273935.s001.pdf]

Consumers Health Forum OF Australia

Curtin University

KITCHEN TABLE DISCUSSION QUESTIONS

Telehealth Experiences & Preferences

Thank you for attending this consumer discussion on telehealth.

Telehealth has become a mainstream tool for accessing healthcare in Australia since the start of the COVID-19 pandemic. Millions of consumers have used their phones, tablets and computers to connect with services across the health system. While most encounters remain at the primary care level such as general practitioners or the family doctor, specialist and allied health services have also become well established and continue to grow.

The success of telehealth means that it's likely to stick around – even after the pandemic ends. CHF is working with the federal Department of Health, the Digital Health CRC and Curtin University to better understand consumer experiences and expectations for telehealth going forward.

Please note that all responses and information used will be deidentified.

Questions for discussion

1. Over the past year have you accessed telehealth services via phone, video, or similar? If not, what was the reason for not doing so?
2. In your experience what would you identify as the strengths of telehealth so far?
3. What about the weaknesses?
4. How would you describe the choices available for telehealth? Did you freely choose between video or telephone for example? If not, what choices were offered to you and by who?
5. Do you feel that telehealth provides you the same quality and experience of care that you receive when seeing a clinician face-to-face?
6. Are there circumstances where telehealth is preferable or more appropriate than to face to face consultations? What would these circumstances be?
7. What concerns (if any) do you have about the privacy of telehealth?
8. Have clinicians discussed privacy issues with you?
9. Would you be willing to pay the same amount (or more) for a telehealth consultation as you would for a face-to-face consultation?
10. Looking into the future, would like to see more telehealth? If so, what could be improved?
