# Supplementary material for: Consumer preferences, experiences, and attitudes towards telehealth: Qualitative evidence from Australia
Source: PLoS One. 2022 Aug 31;17(8):e0273935. doi: 10.1371/journal.pone.0273935 (PMC9432716; doi:10.1371/journal.pone.0273935)
Supplement: Attachment — Submitted filename: Response to Reviewers.docx [file pone.0273935.s002.docx]

# Response to Reviewers

Dear the Editor and Reviewers,

We, the authors, would like to thank both the Editor and Reviewers for the time taken to thoroughly review and provide feedback to our manuscript. We have found this to be incredibly useful and enabled the manuscript to be developed into a more precise and well-thought through version.

Thank you for the opportunity to make these revisions to our manuscript and resubmit a version that addresses each of the points raised during the review process. The table below shows each point raised by the Editor and each Reviewer, and how we have responded to each point. The line numbers stated are for the marked-up copy of the manuscript, so will differ to the unmarked version.

Thank you again for the invaluable feedback and time given, and for considering our manuscript for the PLOS ONE journal.

Yours sincerely,

Kaylie Toll

On behalf of the authorship team

| **Editor** | **Response** |
| --- | --- |
| 1. PLOS ONE's style requirements | Manuscript has been adjusted to meet style requirements and uploaded into editorial manager |
| 2. Please include your tables as part of your main manuscript and remove the individual files | Tables have been included in manuscript and removed individual files. Additional supplementary file has been included (KTD questions) |
| 3. ‘Funding Information’ and ‘Financial Disclosure’ sections do not match | This has been adjusted to more accurately represent the funding information and to be consistent throughout:  This research was part of a larger study supported by the Australian Government Department of Health, Health Economics and Research Division, and the Digital Health CRC Limited (DHCRC), funding number DHCRC0161. The DHCRC is funded under the Commonwealth’s Cooperative Research Centres (CRC) Program. The Department of Health had no role in study design, data collection and analysis, or preparation of the manuscript. The DHCRC author had involvement in study design and development of the manuscript. Both organizations support our decision to publish. |
| 4. Please state what role the funders took in the study. If the funders had no role, please state: "The funders had no role in study design, data collection and analysis, decision to publish, or preparation of the manuscript."   If this statement is not correct you must amend it as needed.  Please include this amended Role of Funder statement in your cover letter | Thank you for recognizing this missing statement. We have included the funding statement as a whole above. We have also included this amended statement in our revised cover letter. |
| 5. you have provided additional information within the Acknowledgements Section that is not currently declared in your Funding Statement. Please remove any funding-related text from the manuscript and let us know how you would like to update your Funding Statement.   Please include your amended statements within your cover letter; we will change the online submission form on your behalf. | We have removed the additional information from the acknowledgement section and all funding-related text from the manuscript.  We have included the amended funding statement in our revised cover letter. Thank you for changing the online submission form on our behalf. |
| 6. In your Data Availability statement, you have not specified where the minimal data set underlying the results described in your manuscript can be found.   All PLOS journals require that the minimal data set be made fully available. For more information about our data policy, please see <http://journals.plos.org/plosone/s/data-availability>.  Please upload your study’s minimal underlying data set as either Supporting Information files or to a stable, public repository and include the relevant URLs, DOIs, or accession numbers within your revised cover letter. | Thank you for highlighting this. We have realized we had not provided the KTD interview questions to allow for replication of the study. These are now included as a supplementary file.  All other data, demographic data and relevant consumer quotes, have been provided within the manuscript, but due to ethics and data governance policies we are not able to provide the interview transcripts. |
| 7. Update Cover Letter with changes | The cover letter has been revised with requested changes (listed in each point) and will be uploaded upon submission |
| 8. While revising your submission, please upload your figure files to the Preflight Analysis and Conversion Engine (PACE) digital diagnostic tool, <https://pacev2.apexcovantage.com/>. | Figures 1 and 2 have been uploaded to the PACE digital diagnostic tool and resubmitted in the editorial manager. |
| **Reviewer 1** |  |
| 1. Could you elaborate if conducting a focus group in a language other than English was intended? | We have amended the manuscript text to include the following on line 181:  *All but one KTD session was conducted in English. Whilst we did not target non-English-speaking focus groups, in selecting KTD hosts we particularly set out to ensure diversity in terms of the potential hosts’ community links. One host had strong links to the Japanese community thus contributing to inclusion of participants from culturally and linguistically diverse communities. Because of the variability in English language proficiency of participants the KTD was undertaken in Japanese. The host translated the outcomes into English.* |
| 2. What were the experiences of the CALD group in accessing telehealth and were there barriers due to language?   Could you elaborate on whether telehealth was conducted with clinicians that spoke the same language as participants or with interpreters or caregivers? | We have added the following sentence to provide further clarification against the first point.  Added Line 372 - *Some participants who spoke English as a second language reported discomfort when conversing over the phone and felt telehealth exacerbated communication issues. To compensate for language limitations, these participants reported often relying on visual information (such as viewing of test results), but the lack of this kind of visual informational aid when using the telephone contributed to a reduction in the level of understanding. Video consultations may help to improve these situations, but some participants reported face-to-face contact was still preferred for optimal communication.*  In relation to the second point participants did not elaborate on whether clinicians spoke the same language as patients, but the indication is the Clinician spoke in English and there was no interpreter available. Line 378: One suggestion is to explore how telehealth could be used to link consumers to practitioners that speak their language. |
| 3. It is noted that face-to-face consultations were preferred by participants when physical examinations were required. Was there any evidence of physical examinations being conducted via videoconference? Did participants find this of value and did they feel prepared and comfortable to undertake this in their homes? | There was no mention of any physical examinations being conducted via videoconference. Line 259: *Participants reported that the lack of ability to do a physical assessment is often perceived as the inability to treat the consumer comprehensively, holistically or with a preventative focus, such that important things could be missed that may otherwise be detected face-to-face.* |
| 4. Is it worth commenting on the under representation of men as a study limitation? | We have added a clearer limitations subheading and added the following sentence on line 660:  *The majority of participants were female (79%), thus there is an underrepresentation of males in the study sample* |
| 5. Minor typographical errors for review. Line 66: 'response' spelt incorrectly. Lines 72-76: sentence is difficult to read and would be helped if simplified? Line 167: missing 'to' after responses? Lines 211-213: colon after Sydney should be a semicolon or comma? Line 227: missing closed bracket after physiotherapy? | Thank you to the reviewer for highlighting these minor issues, the following action has been taken.  Line 66 (now 69): corrected  72-76 (now line 79): revised:  *The Australian Government has enabled provision of both phone and video consultations, with video being the preferred substitution for face-to-face when telehealth is clinically appropriate.*  *However, uptake for phone consultation is higher and it is unknown if these usage rates are due to consumer preference or the availability given to consumers.*  167 (now 179): corrected  211-213 (now 239): corrected  227 (now 256): corrected |
| **Reviewer 2** |  |
| 1. Research methodology - Within qualitative, there are number of methodologies (grounded theory, phenomenology, ethnography etc). What is the qualitative methodology for this study? | The following text has been added in response to the methodology question (see line 192)  *The qualitative analysis employed a combination of inductive (generating new knowledge) and deductive (testing theories) techniques* |
| 2. Kitchen Table Discussion – I have not heard of this before but sounds very interesting. Why was this chosen as the method of data collection? Has this been tried before in other similar research? | The methods section lines 111-119 outline the response to this question and include:  *Kitchen table conversations have been used in various settings and contexts such as local government and political campaigns (18-21). The Kitchen Table Discussion (KTD) methodology, pioneered first in the consumer sector by Health Consumers Queensland (22), supports the training of consumer facilitators (hosts) with a strong community network to conduct focus groups with peers. The sessions involved discussing consumer attitudes and experiences via a semi-structured focus group format. The aim of this approach is to enable informal and relaxed dialogue, so health consumers, carers and community members, who do not ordinarily participate in healthcare consultations, are able to have their say in a safe and supportive environment.* |
| 3. Sampling – How were the participants identified and recruited? There is no reference in this section. Sampling framework needs more details and references. | We have made reference (line 124) to the sampling methods used for hosts:  *The KTD hosts were selected via purposive sampling through a widely disseminated expression of interest process coordinated by The Consumers Health Forum of Australia (CHF). Applications were assessed on the basis of a potential host’s community links and the level of diversity this could deliver in terms of KTD participants*.  And a separate section (detail) for participants (line 137):  *Participants were recruited by their hosts through non-probability convenience sampling with each host inviting up to 10 community members via their own local community connections, to attend either online or in-person group discussions.* |
| 4. Recruitment – There are two groups here from what I understand. There are the “hosts” and “participants”. It would be good to break the recruitment strategies into these two groups and report it as such. For example, how did the hosts source the contact details of the participants? How did the researchers ensure there was enough coverage of participants? If the host and participants were social acquaintances, how was coercion avoided? | The reviewer is correct there are two distinct groups. We have separated these groups in the manuscript and provided more detail on recruitment strategies. See above text.  *Hosts received: training via a Zoom videoconference; a comprehensive host guide; questions to ask participants during the discussion; a feedback report template; support from CHF to ensure a successful session; and appropriate remuneration in recognition of their time.* |
| 5. Data collection – So the hosts recorded participant responses each question onto template and then it was audiorecorded and transcribed? Is that right? If so, why did you collect the same data in two different ways? | A decision to use two data collection methods for KTD discussion was chosen due to potential issues with digital recording including:  All participants in a focus group needed to consent to being audio recorded, if one participant didn’t consent then no recording could go ahead  In case of technical issues with recording that impacted on the recording taking place or the quality of the recording |
| 6. Who did the transcription of the interviews? | *A member of the research team transcribed all electronic recordings* – a sentence has been added to the manuscript to clarify this see line 183 |
| 7. How was observation data managed? | No observation data was collected. |
| 8. Rigour –It would be useful to outline clearly the strategies used and how this contributed to the rigour of the data collection and analysis. This is not clearly articulated. | In terms of data collection all hosts had training in hosting a KTD, had hosts guides and a reporting template.  In terms of data analysis, the study followed a process as outlined by Braun and Clarke this is referenced in the manuscript from line 192.  *The qualitative analysis employed a combination of inductive (generating new knowledge) and deductive (testing theories) techniques (23). All transcribed session data, report templates and demographic data forms were transferred to researchers electronically, making sure all data was de-identified. Data were analysed using thematic analysis and identified key themes.*  *The phases of thematic analysis outlined by Braun and Clarke (24) were applied by the research team, in the six following steps:*   - *Familiarisation with the data* - *Generation of initial codes* - *Searching for themes* - *Reviewing themes* - *Defining and naming themes* - *Producing the report.*   *Data were analysed sequentially, for each of the 10 questions that comprised the semi-structured focus group interview template followed by the hosts.*  *For each question, an independent researcher analysed the transcriptions and host reports through an open-coding method to generate initial codes. These codes were then applied, reviewed, and categorised into sub-themes, each then defined and labelled with a collective theme that described the core concept for each question.* |
| 9. Data collection –Was there any follow up to clarify any issues? How did member checking actually occur? Again, there needs to be references here to defend your choice. | The data analysis process has been made clearer, with line 210 added:  *To ensure rigour and validity a second researcher independently reviewed the transcripts, the coding and categorisation of subthemes.* |
| 10. Sample size – how was sample size determined? | Our goal was to reach a variety of participants and so a sample size that allowed for a variety of experience and options to emerge. Following the host recruitment, the research team believed that aiming for between 5-10 participants across the KTD would allow us to obtain saturation. |
| 11. Sometimes you use the word “interview” and sometimes it is focus group. Please be consistent. | We have corrected this inconsistency |
| 12. How did you ensure rigour and trustworthiness? | The data analysis process has been made clearer, with line 210 added:  *To ensure rigour and validity a second researcher independently reviewed the transcripts, the coding and categorisation of subthemes.* |
| 13. “The interviews opened by asking respondents about the modality of telehealth services and which if any services are preferred for telehealth” – this is Method right? Not results. | We have edited the manuscript and removed the sentence out that refers to the methods from the results section. |
| 14. “Telehealth consults were provided across the public, private and community services sector, with the majority conducted over the phone.” – where is the data to back this up in text? | The data is based on participant responses to the question on modality included in the KTD interview schedule.  Line 231: *More than two thirds of participants reported using telehealth in the past (77%; n=69), with nearly three quarters (69%) conducted over the phone versus videoconference (31%).* |
| 15. For each quote, it is important to provide some information about the person who reported it. You could include a pseudonym or other characteristic as it would add weight to your results and show the reader who said what. | We decided not to provide such detail due to potential re-identification. |
| 16. Need a section on limitations. | We have added a clearer limitations subheading to incorporate the points above |
| 17. Implications for practice and research – Based on the findings from this research, what are the implications for practice and research? | We have added a clearer policy, practice and research implications subheading to incorporate the points in a clearer fashion. |
