# Supplementary material for: Consumer preferences, experiences, and attitudes towards telehealth: Qualitative evidence from Australia
Source: PLoS One. 2022 Aug 31;17(8):e0273935. doi: 10.1371/journal.pone.0273935 (PMC9432716; doi:10.1371/journal.pone.0273935)
Supplement: Attachment — Submitted filename: Response to Reviewers.docx [file pone.0273935.s003.docx]

# Response to Reviewers

Dear the Editor and Reviewers,

We, the authors, would like to thank both the Editor and Reviewers for the time taken to review our previous responses and provide feedback to our manuscript. We have found this to be incredibly useful and enabled the manuscript to be developed into a more precise and well-thought through version.

Thank you for the opportunity to make these revisions to our manuscript and resubmit a version that addresses each of the points raised during the review process. The table below shows each point raised by the Editor and each Reviewer, and how we have responded to each point. The line numbers stated are for the tracked changes copy of the manuscript, so will differ to the unmarked version.

Thank you again for the invaluable feedback and time given, and for considering our manuscript for the PLOS ONE journal.

Yours sincerely,

Kaylie Toll

On behalf of the authorship team

| **Journal requirements** | **Response** |
| --- | --- |
| 1. Please review your reference list to ensure that it is complete and correct. If you have cited papers that have been retracted, please include the rationale for doing so in the manuscript text, or remove these references and replace them with relevant current references. Any changes to the reference list should be mentioned in the rebuttal letter that accompanies your revised manuscript. If you need to cite a retracted article, indicate the article’s retracted status in the References list and also include a citation and full reference for the retraction notice. | We have checked our reference list and had added one reference in the previous response that was not present in the initial manuscript.  The new reference is listed as *35. Hall Dykgraaf S, Desborough J, de Toca L, Davis S, Roberts L, Munindradasa A, et al. "A decade's worth of work in a matter of days": The journey to telehealth for the whole population in Australia. Int J Med Inform. 2021;151:104483.*  This provided additional evidence to our findings, *“Other studies also highlighted that the availability and modalities of telehealth are often provider driven rather than consumer led”.* (line 742)  We apologise for not highlighting this on our previous response. |
| **Reviewer 1** |  |
| 1. All comments have been addressed | Thank you again for your time reviewing our responses and changes to the manuscript. |
| **Reviewer 3** |  |
| The authors have not made all data available (but have explained the reasons for this adequately). I felt the manuscript was well written and in the main, the previous reviewer comments had been dealt with adequately. Having not seen the manuscript prior to its resubmission following responses to peer review, I have not conducted a full, fresh review, but restrict my comments to the extent to which I feel that previous reviewer comments have been dealt with. I suggest some further minor amendments. | We would like to thank the reviewer for their time and feedback, recognising the difficulty coming in at a later date, but thank them for seeking clarity on the previous reviewer’s points. This has helped the authors make the following points more robust. |
| 1. Reviewer 2 noted the possibility of coercion if participants were previously known to hosts or were social acquaintances. The authors did not address this point in their response but it is an important one that has bearing on the rigour, reproducibility and credibility of the data, so I would ask that the authors include a specific comment within their methodology on this. It is likely that most of the participants knew the hosts because the hosts took the lead on sampling and recruiting participants from within their networks. Allied to this - what influence (if any) did the researchers have on the diversity of participants that the hosts recruited e.g. was there a sampling frame that hosts were required to use, or did they have complete carte blanche over who they recruited to be in the KTD? | We have added an additional line 124: “*This training and guidance was designed to emphasise their role as a moderator, and not a participant or responder.”*  Plus, additional text on line 130-136: “*The participants were community acquaintances of the host, enabling health consumers, carers and community members who do not ordinarily participate in healthcare consultation to have their say in a safe and supportive environment. The recommendations to hosts for sampling from the host guide was as follows: “The group of people should reflect a wide diversity of ages, cultures, and health experiences. Please choose your participants mindfully, ensuring there is no conflict of interest, or you feel coerced in any way.”* |
| 2. It is not clear why the issues with data recording arose in the first place. Usually, if a participant does not consent to being recorded, they are in essence not consenting to the study and ought to take no further part in it. It is strange that the authors chose instead to not record the entire session simply because one individual may not have wanted to be recorded. This may have implications for the quality of data from the sessions that were not recorded, and I would like to see more reflection on this approach (and its potential consequences) in the paper. | Consent information has been updated on line 143: *“We adopted a two-stage consent process, participants were able to consent to the study and consent to the audio recording separately. Not consenting to the recording did not exclude participants from the study.”*  We have also updated the manuscript text in order to make this issue clearer, line 180-185:  *“The sessions were conducted between 10^th^ and 28^th^ May 2021 and lasted approximately 90 minutes each and were either recorded and transcribed, or scribed during the session. The host guide recommended to audio record each session to allow better engagement in discussion and support report template completion. Some participants did not consent to the audio recording, which did not exclude them from the study, and other groups encountered technical difficulties.”*  Line 186-189 has been updated for clarity: “*The hosts completed a written report of participant responses based on a template and attached demographic data forms, participant registration sheets and completed consent forms, and sent to the research team. A member of the research team transcribed all available electronic recordings.”* |
| 3. Whilst the authors have addressed a point about the data analysis process from the perspective of dual coding from a second researcher, the authors did not address the important point from reviewer 2 about member checking e.g. were hosts and/or participants given the transcripts so that they had the opportunity to amend anything they said or withdraw any of their statements? If not, why not?   Similarly, rigour and trustworthiness can be broader than simply having dual review of transcripts/coding (reviewer 2 point 12). I would invite the authors to consider how they ensured rigour/trustworthiness in a broader sense than just in the data analysis stage. | Along with point 2 above, we have added additional text in line 176-179: Each participant was given a chance to answer each question, using their own words, *including real-time check back by hosts to ensure the information captured was true to the respondent.* Participants were invited to make any additional comments at the end of each session or email them following the session”.  The following text has also been included to address this point on line 699:  “*A further limitation was the reliance on Hosts to take notes during some sessions when audio recording was not an option.”* |
| 4. The authors chose not to add any information about the person saying a quotation because of the possibility of anonymity being broken. This is a legitimate concern but would actually not preclude the authors adding some very brief information on an individual that would not break participant confidentiality, such as gender, broad age (e.g. under 50, above 50), some sense of urbanity/rurality (given that this is one of the characteristics the authors surmise may make a difference to patient receptiveness to telehealth), or past use of telehealth yes/no. I would maintain that having some information about the person who reported a quotation is still very important, and can be done sensitively without any risk that a participant would be identified from the manuscript. I would ask the authors to reconsider their decision on this point. | We agree that it would be beneficial to provide brief information on participant quotes that would not breach confidentiality.  We have updated the methods section on line 218 to include the following: *“Quotes are used to demonstrate findings in each theme from the voice of the participant. Each quote includes the participant allocated identification number, 1-90, location based on the Modified Monash Model and past telehealth usage. For example: P5, major city, used telehealth.”* |
